# Genes Contributing to the Development of Alcoholism: An Overview

**DOI:** 10.35946/arcr.v34.3.08

**Published:** 2012

**Authors:** Howard J. Edenberg

**Affiliations:** **Howard J. Edenberg, Ph.D,***is a Distinguished Professor and Chancellor’s Professor in the Department of Biochemistry and Molecular Biology and the Department of Medical and Molecular Genetics, Indiana University School of Medicine, Indianapolis, Indiana.*

**Keywords:** Alcoholism, alcohol metabolism, genetic basis of alcoholism, genetics, genetic factors, human studies, case–control studies, family studies, candidate gene analyses, genome-wide association studies, alcohol dehydrogenase, aldehyde dehydirogenase

## Abstract

Genetic factors (i.e., variations in specific genes) account for a substantial portion of the risk for alcoholism. However, identifying those genes and the specific variations involved is challenging. Researchers have used both case–control and family studies to identify genes related to alcoholism risk. In addition, different strategies such as candidate gene analyses and genome-wide association studies have been used. The strongest effects have been found for specific variants of genes that encode two enzymes involved in alcohol metabolism—alcohol dehydrogenase and aldehyde dehydrogenase. Accumulating evidence indicates that variations in numerous other genes have smaller but measurable effects.

A major goal of genetic research into alcoholism and related traits is to better understand the biology underlying this disease by identifying specific genes in which variations contribute to a person’s risk of developing the disease and then examining the pathways through which these genes and their variants affect the disease. Researchers hope to use this knowledge to develop new, more effective, and more targeted treatment and prevention strategies. For complex diseases such as alcoholism, however, this is a very difficult endeavour. There is no one gene (or several) whose particular variants “cause” the disease. Instead, variations in many, and perhaps hundreds, of genes likely have a small but measurable influence on disease risk that ultimately adds up to a substantial impact. Moreover, the impact of any one gene variation depends both on the individual’s genetic background (i.e., other genetic variations the person carries) and on the environment. These factors further complicate the identification and confirmation of the role of any one gene. This overview briefly summarizes some of the strategies that can be used to identify specific gene variants that influence the risk of alcoholism and reviews some of the findings obtained to date, setting the stage for the following articles in this Special Section.

## Strategies for Identifying Genes Associated With Alcoholism Risk

Several study designs—including case–control studies, population studies, and family studies—have been used to test whether a specific gene or gene variant affects risk for a disease (for more information, see the article by Foroud and Phillips, pp. 266–272). There are advantages and disadvantages to each approach. For example, it is much easier to collect individual cases (i.e., people with alcoholism) and control subjects (i.e., nonalcoholic people) or samples of the general population than it is to recruit family samples. Moreover, family studies require more effort to determine the participants’ genetic makeup (i.e., genotype), because even with the simplest type of family study, genotypes must be determined for sets of three people (e.g., two parents and an affected child) rather than just for individual case and control subjects. On the other hand, family studies avoid the problem of incomplete ethnic/population matching^1^ that can confound case–control studies. Furthermore, family studies can be more powerful than case–control studies if different variants (i.e., alleles) of the same gene affect a given trait in different families, because multiple families can show an effect of that gene despite not sharing the same alleles. In addition, broad regions of the genome generally are inherited within a family, increasing the sensitivity of the approach to detect an effect; however, the tradeoff is that for the same reason, family studies have less resolution to identify the specific allele(s) involved. When both types of studies point to the same genes, however, it provides additional evidence for the involvement of these genes.

There also are two strategies for deciding which genetic variations to test. The first involves focusing the testing on specific genes that are selected on the basis of their physiological roles or their reported involvement in related traits. These so-called candidate gene studies have been fruitful in alcohol research. For example, they led to strong evidence that genes that encode the two main enzymes involved in alcohol metabolism—alcohol dehydrogenase (ADH) and aldehyde dehydrogenase (ALDH)—affect risk, which will be discussed in the next section. Some of these studies, particularly the earlier ones, only have assessed a single allele of a candidate gene, whereas in other studies a set of alleles was chosen to provide information on most of the common variations in the gene.

The other approach is the genome-wide association study (GWASs), which examines a large set of variations in many genes distributed acrosss the entire genome. Each of these variations involves only a single DNA building block (i.e., nucleotide), and they therefore are known as single nucleotide polymorphisms (SNPs). Because such studies may test 1 million SNPs (although not all of these are independent of each other), this can involve 1 million separate tests for association, and therefore an increased chance of false positives if the *P* value is not adjusted. In fact, to guard against false-positive associations between a SNP and a given trait, the current view is that a *P* value smaller than 5 × 10^−8^ is required. However, some think that this requirement may be too stringent, because many genes and interactions are expected to play a role in a complex disorder such as alcoholism. In practice, the GWASs approach requires very large samples or the aggregation of many studies, and most genome-wide studies on alcoholism do not have sufficient statistical power to detect the small effects expected for individual genes. In contrast, the statistical penalty for multiple testing is reduced greatly with candidate gene studies because these studies test specific hypotheses, and most of the specific genes thus far associated with alcoholism have been identified in candidate gene studies.

## Genes Implicated in Alcoholism Risk

### Genes Encoding Enzymes Involved in Alcohol Metabolism

The genes most strongly implicated and best characterized are those encoding the key enzymes of alcohol metabolism, ADHs and ALDHs. A variation in the gene encoding mitochondrial ALDH2 (i.e., the ALDH2*2 allele)^2^ renders the resulting enzyme nearly inactive so that the levels of acetaldehyde circulating in the body increase substantially when alcohol is consumed. This acetaldehyde accumulation underlies the strongly aversive flushing reaction (for more information, see the article by Hurley and Edenberg, pp. 339–344). An abundance of physiological and molecular data has demonstrated how this allele affects alcohol metabolism. People carrying a single copy of the ALDH2*2 allele in their genome are highly protected against alcoholism. Yet this strong effect still can be modified by the environment, as clearly shown by Higuchi and colleagues (1994), who found that the level of protection afforded by ALDH2*2 in the Japanese population dropped significantly with time as the social pressures for drinking increased. People carrying two copies of the ALDH2*2 allele, however, become so ill after consuming alcohol that their risk of becoming alcohol dependent is near zero.

Another well-studied gene variant concerns the gene encoding the ADH1B enzyme. This ADH1B*2 allele, which encodes an enzyme with higher activity, also is highly protective against alcoholism. Detailed molecular studies of this allele have been carried out, and although physiological studies did not detect the same dramatic rise in circulating acetaldehyde as with the ALDH2*2 allele, the ADH1B*2 allele has a similar effect on risk (see the article by Hurley and Edenberg, pp. 339–344).

Gene variants related to alcoholism risk that are present in a population at low frequency are difficult to detect because the number of people who need to be genotyped increases dramatically. For example, the ALDH2*2 allele that has such a strong effect is essentially absent in many areas of the world and therefore is not detected in studies of most populations. However, the effect of ALDH2*2 on risk for alcoholism is easy to detect even in relatively small studies of populations in which it is common, for example in China and Japan. Likewise, the ADH1B*2 allele, which is very common in East Asia and relatively common in the Middle East, is relatively uncommon (i.e., generally has allele frequencies of less than 4 percent) in most other places. Thus, it is easy to detect the effect of ADH1B*2 even in small studies of Asian populations but much more difficult in other populations (Li et al. 2011). However, a recent study in which the ADH1B*2 allele was genotyped in several thousand people of European ancestry showed a highly significant protective effect, comparable in magnitude to that in Asians (Bierut et al. 2012). Therefore, the exact population studied, the size of the study, and the exact trait studied all are important to consider when comparing results.

To date, no other gene has been identified that harbors variations with effects on alcohol dependence as strong as those of the ALDH2*2 or ADH1B*2 alleles. Other alleles of the *ADH* and *ALDH* genes also have been reported to affect risk; however, these effects are much smaller and are not detected in all studies. For example, several studies found other variations in and near the *ADH1B* gene, as well as in or near the *ADH4*, *ADH1C*, *ADH5*, *ADH6*, and *ADH7* genes that affect risk for alcoholism or the level of alcohol consumption (see the article by Hurley and Edenberg, pp. 339–344). Importantly, many of these other alleles do not affect the structure of the encoded protein but probably act by altering the level of gene expression. Therefore, it is important to also study the effects of various alleles on gene regulation. Another complication is that many *ADH* and *ALDH* genes are located on the chromosomes in clusters, and many nearby variations therefore are inherited together (i.e., in haplotype blocks). As a result, researchers cannot always determine which allele in such a block has the observed effect or whether several alleles might be involved. Studies in populations with different genetic histories can help disentangle the roles of individual alleles, although environmental differences between these populations might complicate the analyses.

### Genes Encoding Other Proteins

Other gene variants also have been associated with the risk for alcoholism, including genes encoding many of the subunits of a receptor for the brain signaling molecule (i.e., neurotransmitter) γ-aminobutyric acid (GABA). This GABA_A_ receptor consists of five subunits, and studies have found that certain alleles of several subunit genes can influence the risk for alcoholism and other addictions, as well as of conduct disorder symptomology and can modify electrophysiological traits related to these disorders. For example, many (although not all) studies have implicated alleles of the *GABRA2* gene, which encodes the α2 subunit of the GABA_A_ receptor, in alcoholism risk (for more information, see the article by Borghese and Harris, pp. 345–353). Other GABA_A_ receptor subunit genes also have been implicated, including *GABRG1*, *GABRA1*, *GABRG3*, *GABRR1*, *GABRR2*, and *GABRB3*. Both physiological and molecular evidence indicates that GABA_A_ receptors are affected by alcohol and participate in many processes relevant to addiction, and studies in rodents have provided further evidence of this involvement. Overall, however, the effects even of the best studied of these genes, *GABRA2*, seem to be small. Although some GWASs have shown nominally significant support, genes encoding GABA_A_ receptor subunits generally have not been among the top genes identified by GWASs.

Variations in many other genes also have been implicated in contributing to alcoholism risk. Among the genes and pathways highlighted in this brief section are genes involved in the immune system, including nuclear factor-κB–related genes (see the article by Crews, pp. 355–361), the circadian system (see the article by Sarkar, pp. 362–366), and genes whose function is not yet clear (see the article by Buck and colleagues, pp. 367–374). Other genes that also have been identified encode components of the neurotransmitter systems using dopamine, endogenous opioids, serotonin, and acetylcholine; nicotinic receptors; and a hormonal system known as the hypothalamic–pituitary axis. This list continues to grow as more GWASs are completed and analyzed.

## Conclusions and Outlook

Although studies in recent years have identified a plethora of genes that may play a role in determining risk of alcoholism, much work remains to be done. For example, many genes have been reported in only one study. Therefore, it will be critical to confirm these associations in additional studies. A failure to replicate the initial findings may not always disprove the association but may result from differences in the genetic background of the study participants, the environment, or the study design (e.g., differences in the definition of alcohol dependence). Beyond replication, the exploration of which specific aspects of the alcoholism phenotype each involved gene affects and which other diseases or traits may be influenced by it is essential. Moreover, it will be equally important to determine the potential underlying mechanisms through functional studies, including the use of animal models, particularly those in which candidate genes or alleles are introduced into the organism (i.e., knocked-in). Although much work remains to be done, researchers already have made substantial progress. New technological developments that allow for faster and more complete genotyping and sequencing will accelerate progress, as will technical developments allowing targeted overproduction or inactivation of genes in animal models.

## Footnotes

1 This means that the samples of case and control subjects may not be sufficiently matched with respect to such factors as ethnicity or other population characteristics, which influence the prevalence of many gene variants or other factors that also may influence alcoholism risk.

2 By convention, gene names in animals are written in uppercase and lowercase and italicized. Gene names in humans are written in all caps and are italicized, whereas the acronyms for the encoded proteins are all caps but not italicized.
